# Reflectometers for Absolute and Relative Reflectance Measurements in the Mid-IR Region at Vacuum

**DOI:** 10.3390/s21041169

**Published:** 2021-02-07

**Authors:** Jinhwa Gene, Min Yong Jeon, Sun Do Lim

**Affiliations:** 1 Institute of Quantum Systems (IQS), Chungnam National University, Daejeon 34134, Korea; genejh@cnu.ac.kr (J.G.); myjeon@cnu.ac.kr (M.Y.J.); 2 Department of Physics, College of Natural Sciences, Chungnam National University, Daejeon 34134, Korea; 3 Division of Physical Metrology, Korea Research Institute of Standards and Science, Daejeon 34113, Korea

**Keywords:** mid-infrared, total reflectance, metrology, primary standard, 3rd Taylor method

## Abstract

We demonstrated spectral reflectometers for two types of reflectances, absolute and relative, of diffusely reflecting surfaces in directional-hemispherical geometry. Both are built based on the integrating sphere method with a Fourier-transform infrared spectrometer operating in a vacuum. The third Taylor method is dedicated to the reflectometer for absolute reflectance, by which absolute spectral diffuse reflectance scales of homemade reference plates are realized. With the reflectometer for relative reflectance, we achieved spectral diffuse reflectance scales of various samples including concrete, polystyrene, and salt plates by comparing against the reference standards. We conducted ray-tracing simulations to quantify systematic uncertainties and evaluated the overall standard uncertainty to be 2.18% (*k* = 1) and 2.99% (*k* = 1) for the absolute and relative reflectance measurements, respectively.

## 1. Introduction

Spectral diffuse reflectance in the mid-infrared (MIR) region is now of great interest in many applications such as thermal imagers, solar panels, and some optical techniques for military camouflage [1,2,3,4]. Thermal imagers measuring temperature require target emissivity as a calibration parameter, where the emissivity can be simply converted from the diffuse reflectance in the MIR region with the help of Kirchhoff’s law [5,6,7]. Passive radiative cooling of solar panels is an easy way to achieve self-adaptive cooling, for which a low reflectance of solar panel surfaces in the MIR region is preferred [8,9]. As for military camouflage, one recent focus is on finding a way to decrease the emissivity (increase the reflectance) of personnel and equipment surfaces in the MIR region for protection from attack at night [10].

Widely used as a primary standard in the visible to near-infrared regions, an integrating sphere-based reflectometer is likewise the most feasible instrument to measure diffuse reflectance in the MIR region [11,12,13,14,15,16]. The working principle of the instrument is either the Sharp–Little method or the third Taylor method in many cases [17,18,19,20,21]. Regardless of which method is taken, two assumptions always follow: that the reflectance of the sample under test (SUT) is the same as that of the integrating sphere inner wall (ISW), and that the exposed SUT area on the ISW is small enough to compare to the area of the entire ISW [22]. However, due to the former assumption, a SUT with unknown reflectance needs a reflectance comparator to measure its reflectance via comparison with a reference standard [23].

In this work, we first built a primary reflectometer for absolute reflectance in the MIR region (2–14 µm) in directional-hemispherical geometry. The primary standard is an integrating sphere-based reflectometer using the third Taylor method. The integrating sphere is made of aluminum (Al), and the ISW is covered by plasma-sprayed Al followed by gold plating to obtain a diffusely reflecting surface in the MIR region [24]. We then made another reflectometer (comparator) for measuring relative reflectance following the same fabrication process as that of the primary standard. Each reflectometer is placed in a vacuum chamber with a Fourier-transform infrared spectrometer (FTIR). The spectral diffuse reflectance scale in the MIR region is achieved using the primary reflectometer, where a homemade diffuse reflector is a transfer artifact used as a reference to calibrate the reflectance of various kinds of SUTs via the comparator. To validate our measurement capability, our reference scale is compared to the one measured by the National Institute of Standards and Technology (NIST), and we find the Korea Research Institute of Standards and Science (KRISS) and NIST scales to agree well, within 1.2%. Lastly, we conducted ray-tracing simulation to quantify systematic uncertainties. The overall standard uncertainty is 2.18% (*k* = 1) for absolute reflectance and 2.99% (*k* = 1) for relative reflectance.

## 2. Primary Reflectometer

**Figure 1:**
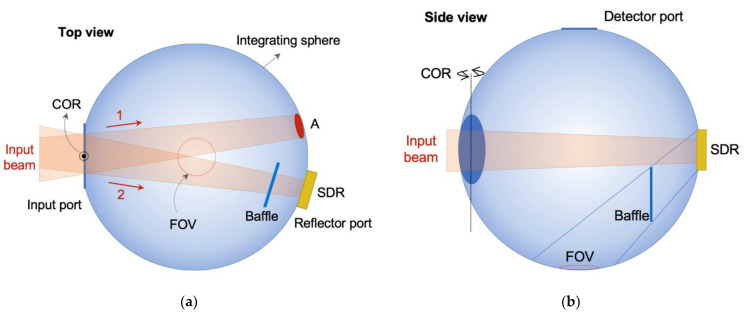
(**a**) Top view and (**b**) side view schematics of the absolute reflectometer integrating sphere. (FOV: field of view, A: area where input beam first illuminates, SDR: standard diffuse reflector, COR: center of rotation).

The reflectometer for absolute reflectance includes an integrating sphere, as shown in the schematic diagrams in Figure 1, with three openings (input, reflector, and detector ports). Absolute reflectance measurement is carried out according to the third Taylor method, as follows: First, light is launched through the input port to the ISW, marked as A in Figure 1a, and the spectral radiance *P*_1_(*λ*) of the area (FOV: field of view) observed by the detector is measured. Then, light is launched to a standard diffuse reflector (SDR) mounted at the reflector port, and spectral radiance *P*_2_(*λ*) is measured.

**Figure 2:**
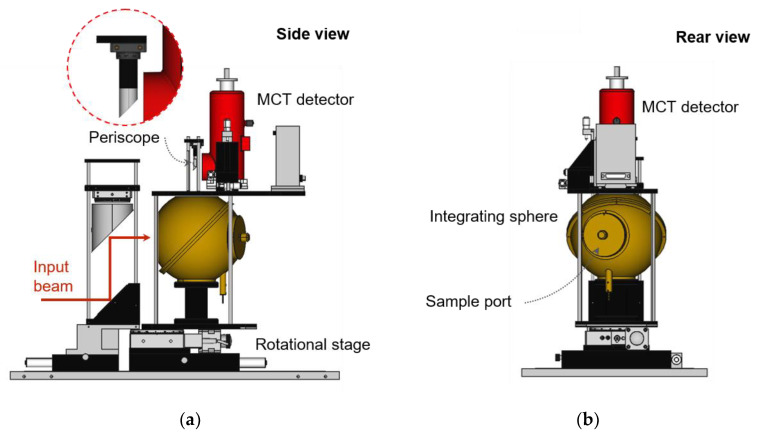
(**a**) Side view and (**b**) rear view schematic images of the absolute reflectometer.

At this stage, the light reflected from the SDR coming directly into the FOV is screened by a baffle, as shown in Figure 1b. The absolute spectral reflectance *R_SDR_*(*λ*) can then be calculated by simple math as follows [22],

(1) $$R_{SDR}\left(\lambda \right)=\frac{P_{2}\left(\lambda \right)}{P_{1}\left(\lambda \right)}\times \frac{A_{0}}{A_{0}-A_{1}-A_{2}}$$

where *A*_0_, *A*_1_, and *A*_2_ are the areas of the entire ISW, the input port, and the detector port, respectively. As mentioned before, Equation (1) is derived based on two assumptions: that the reflectance of the SDR is the same as that of the ISW, and that the exposed area of the SDR is small enough to compare to the entire ISW. We prepared a number of SDR candidates made in the same way as the ISW. Reflection characteristics of their surfaces were tested; see Section 5 for the results. With the integrating sphere, we built a reflectometer for absolute reflectance as shown in Figure 2. The input beam goes into the integrating sphere after two mirrors, a plane mirror and a parabolic mirror with a focal length of 229 mm. The input beam alternately illuminates the wall (A) and the SDR by rotating the integrating sphere at the center of rotation (COR) (Figure 1a). The integrating sphere is installed on a rotational stage with an angle of rotation of ± 8°; thus, the absolute reflectance is measured in 8°/D geometry [23]. The inner diameters of the integrating sphere, the input port, the reflector port, and the FOV are about 150, 43, 25, and 20 mm, respectively. The FOV is defined by the periscope and the window of the MCT detector used. Signals measured by the detector are transformed into spectrally resolved radiance by an FTIR.

## 3. Relative Reflectometer

**Figure 3:**
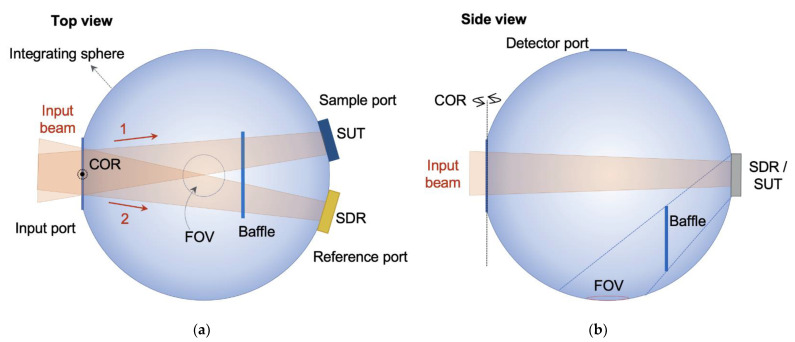
(**a**) Top view and (**b**) side view schematics of the relative reflectometer integrating sphere. (FOV: field of view, SUT: sample under test, SDR: standard diffuse reflector, COR: center of rotation).

Since absolute reflectance measurement can be carried out only for a reflector whose reflectance is the same as the sphere wall, relative reflectance measurement is needed for a reflector with presumably different reflectance from the sphere wall. Figure 3 shows schematic diagrams of the integrating sphere for the measurement of relative reflectance. Compared to the integrating sphere in the absolute reflectometer, the sphere in this case has four openings (input, sample, reference, and detector ports), and the baffle is wider to screen some of the light reflected from both the reference and the SDR directly coming into the FOV, as shown in Figure 3. The procedure for relative reflectance measurement is as follows. First, light is launched to the sample through the input port, as shown in Figure 3a, and the spectral radiance *P_A_*(*λ*) of the area at the bottom of the sphere observed by the detector is measured. Then, light is launched to the SDR mounted at the reference port, and the spectral radiance *P_B_*(*λ*) is measured. The relative reflectance *R*(*λ*) is then calculated as follows [23]:

(2) $$R\left(\lambda \right)=\frac{P_{A}\left(\lambda \right)}{P_{B}\left(\lambda \right)}\times R_{SDR}\left(\lambda \right)$$

**Figure 4:**
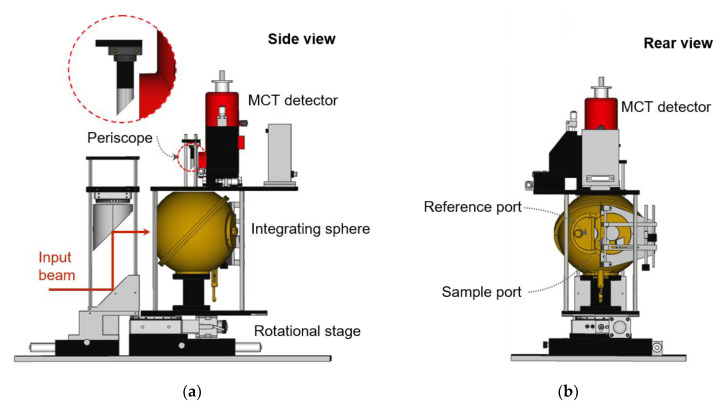
(**a**) Side view and (**b**) rear view schematic images of the relative reflectometer.

With this integrating sphere, we built a reflectometer for relative reflectance, as shown in Figure 4. The input beam again enters the integrating sphere after two mirrors, which are the same as in the absolute reflectometer (a plane mirror and a 229-nm-focal-length parabolic mirror). The input beam alternately illuminates the SUT and the SDR by rotation at the COR (Figure 3), and the relative reflectance is measured in 8°/D geometry [15]. The inner diameters of the integrating sphere, the input port, the sample port, the reference port, and the FOV are about 150, 43, 25, 25, and 20 mm, respectively.

## 4. Overall Measurement System

**Figure 5:**
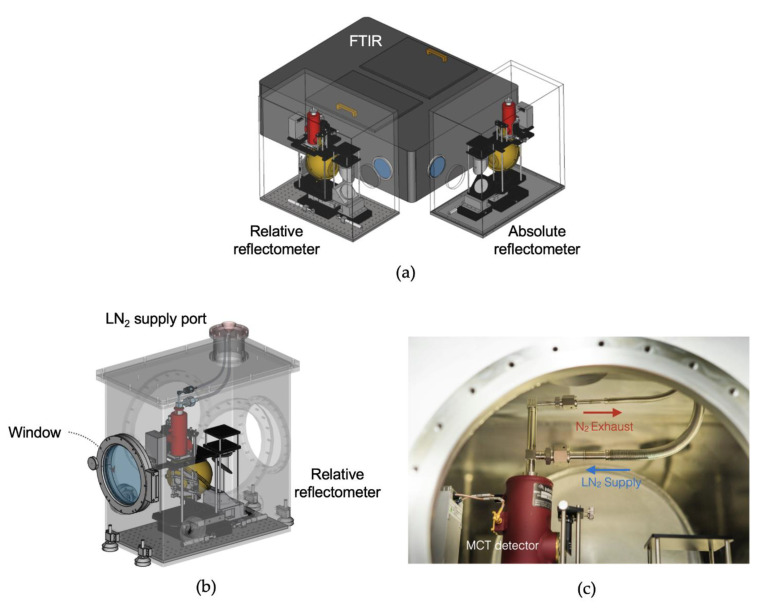
(**a**) Perspective schematic of the reflectometer in the vacuum chamber. (**b**) Schematic of the absolute reflectometer in the vacuum. (**c**) Liquid nitrogen (LN_2_) supply and nitrogen (N_2_) gas exhaust system for the MCT detector.

Optical measurements in the MIR region must always take into account atmospheric molecules in the measurement environment because of their strong spectral absorptions that can distort the measurement results. Purging the measurement system with dry, clean air has been done in reflectance measurements by many previous researchers [23,25,26]. Since even a small portion of molecules unremoved from the system could greatly affect measurement uncertainty, the purging flow in the system has to be designed very carefully. With integrating spheres as in the present work, though, purging is typically ineffective. Therefore, we decided to place the entire measurement system into a vacuum chamber with an FTIR capable of vacuum mode operation (model: Vertex 80v, Bruker). The entire system is shown in Figure 5a. We built the vacuum chambers with stainless steel (SUS304) plates of 20 mm thickness, where each reflectometer is installed inside. The SUT and SDR can be taken in and out through the window, as shown in Figure 5b. Liquid nitrogen (LN_2_) is supplied to the MCT detector through the chamber with a flexible bellows joint; we linked another flexible bellows joint to the MCT detector to lead the evaporated LN_2_ out, as shown in Figure 5c. The pressure was lowered to 10^−3^ bar by a rotary vacuum pump.

## 5. Ray-Tracing Simulation about Systematic Uncertainty of Primary Reflectometer

**Figure 6:**
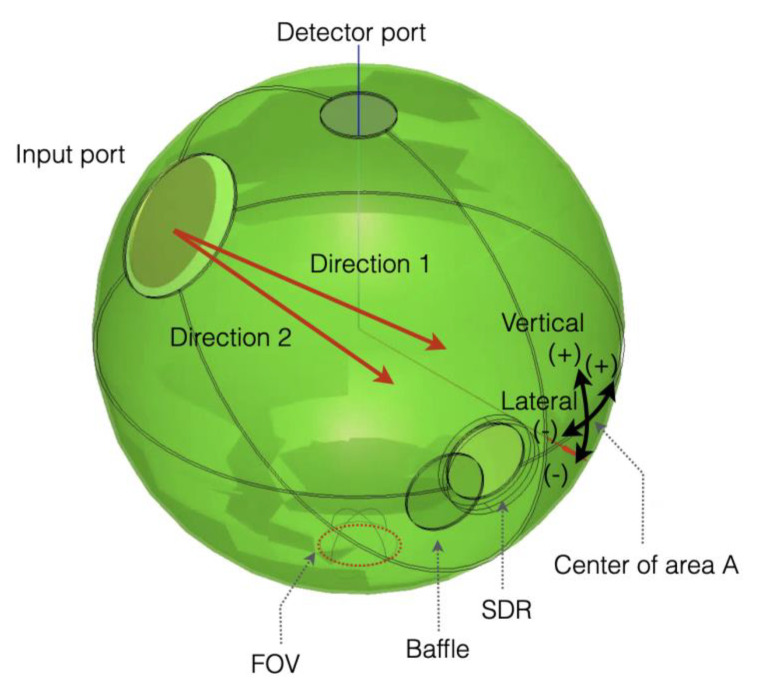
Absolute reflectometer virtually built in 3D space for ray-tracing simulation. The vertical and lateral black arrows at the center of area A show the direction of misalignment in the simulation.

The integrating sphere-based reflectometers here are accompanied by the assumption that the ISW has perfect diffuse reflection (Lambertian reflection) [27]; this, however, is hard to achieve in reality. In particular, realizing Lambertian reflection in the MIR region is more technical than in the visible and near-IR regions where PTFE or BaSO_4_ are widely used [24]. To introduce highly diffusive reflection in the MIR range to the ISW, we applied plasma-sprayed Al to the ISW followed by gold plating. Still, the diffuse reflection from the ISW is not as perfect as PTFE or BaSo_4_. With the fact that imperfect diffuse reflection could be the main source of uncertainty, we conducted ray-tracing simulation using a commercially available tool to quantify the systematic uncertainties arising from the use of an integrating sphere with such low diffusivity. Diffusivity here is defined by the ratio of the power of the diffusively reflected light to the total power of the reflected light [28]. For Lambertian reflection, diffusivity is one, and for perfect specular reflection, diffusivity is zero. The control parameters in the simulations are the diffusivity and reflectance of the ISW. Figure 6 shows a three-dimensional (3D) image of the integrating sphere for absolute reflectance measurement.

**Figure 7:**
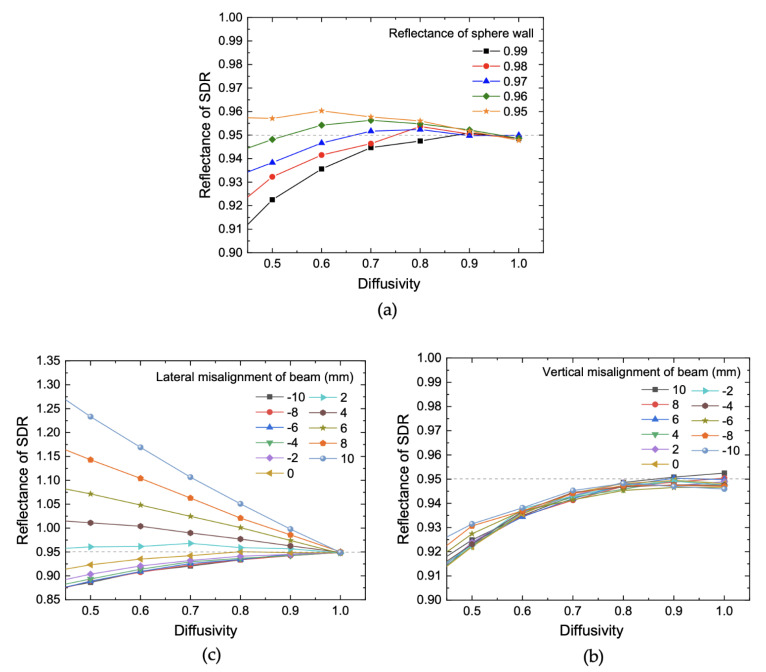
Virtually measured reflectance with the absolute reflectometer with varying system parameters: (**a**) diffusivity and reflectance of the sphere wall, (**b**) diffusivity and vertical misalignment of the incident beam, (**c**) diffusivity and lateral misalignment of the incident beam.

In the simulation, the ISW reflectance is set to 0.95, and the SDR reflectance is calculated from the ratio of the number of rays to the detector to the total number of input rays. We set the number of input rays to 10^8^. Simulations were run for different diffusivities and reflectances of the ISW, while the SDR diffusivity was fixed at 1.0. The calculated SDR reflectance is plotted as a function of diffusivity for different reflectances in Figure 7a. It can be seen that when the ISW has Lambertian reflection (diffusivity is one), the SDR reflectance is calculated to be approximately 0.95, as it was initially set. However, as ISW diffusivity decreases, SDR reflectance becomes unreliable, varying with ISW reflectance [29]. We also investigated the influence that angular tilts of the input beam had on SDR reflectance. Figure 7b,c plots SDR reflectance as a function of vertical and horizontal shifts of the input beam, respectively. It is obvious that a slight shift of the input beam has almost nothing to do with SDR reflectance if the ISW has Lambertian reflection, which is also revealed by the simulation. However, as the ISW diffusivity dips below one, SDR reflectance is under- or over-estimated. This is because some portion of the reflected light off the ISW exits the sphere through the input port before it strikes the detector or strikes the detector before sufficiently reflecting off the ISW. We therefore measured ISW diffusivity to quantify the uncertainties stemming from low ISW diffusivity. The measurement was made for the ISW sample by using 2D bidirectional reflectance distribution function (BRDF) measurement instrument at 2 µm wavelength.

**Figure 8:**
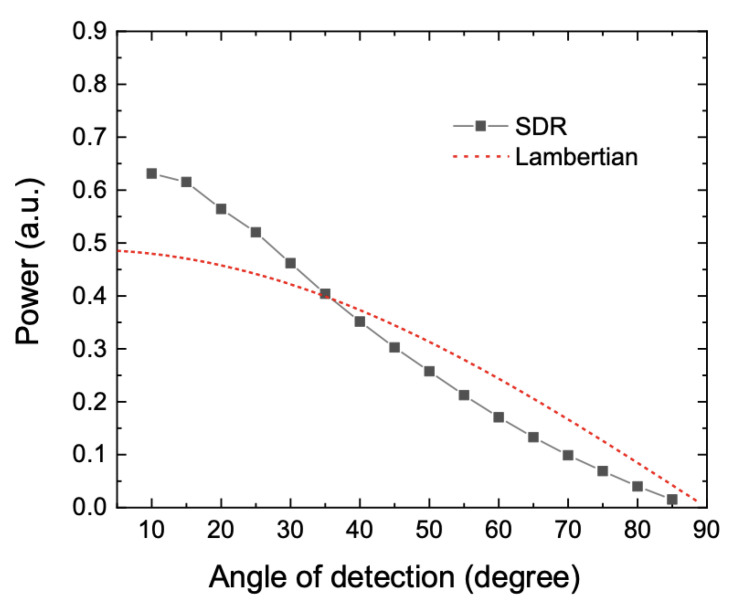
Measured bidirectional reflectance distribution function (BRDF) of the standard diffuse reflector (SDR) at 2 µm wavelength with the same reflection characteristics as the integrating sphere inner wall (ISW).

The sample was illuminated at normal incidence, and the light reflected from the sample was measured with an angle of 10° to 85° from the normal incidence. The distance between the detector, and the sample was 150 mm. Figure 8 shows the BRDF measurement results, from which we calculated ISW diffusivity assuming that the specularly reflected light from the sample is within an angle of 20°.

Calculation was made in 3D reflection configuration, which assessed the ISW diffusivity to be 0.95. Accordingly, systematic uncertainties caused by the 0.95 ISW diffusivity and presumably 4 mm lateral shift were estimated to be about 2% (*k* = 1).

## 6. Reflectance Measurements and Uncertainty Evaluation

**Figure 9:**
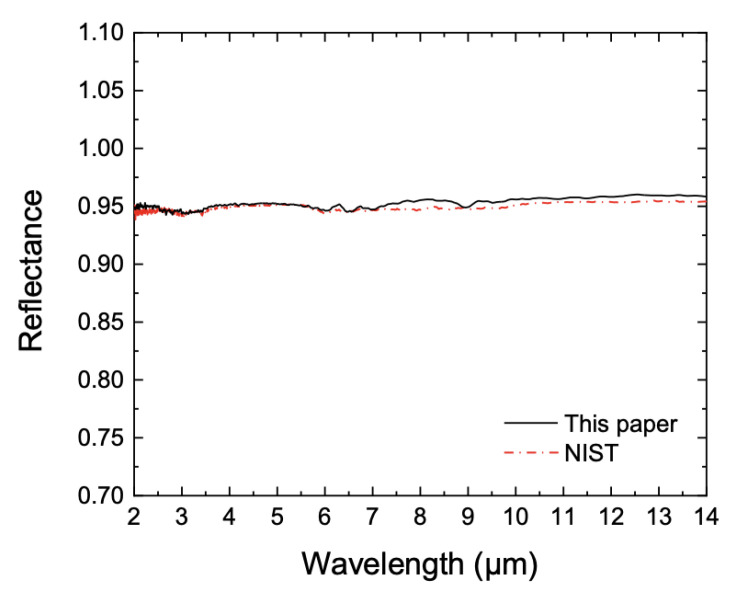
Spectral diffuse reflectance of the homemade SDR (Korea Research Institute of Standards and Science (KRISS)) compared to that by National Institute of Standards and Technology (NIST).

The reflectometer for absolute reflectance in directional-hemispherical geometry operates at vacuum with a pressure of 10^−3^ bar. According to the measurement procedure mentioned in Section 2, the spectral radiance of the FOV was measured when the ISW was illuminated, *P*_1_ (*λ*), and then when the SDR was illuminated, *P*_2_ (*λ*). The measurement was repeated in the sequence *P*_1_ (*λ*), *P*_2_ (*λ*), *P*_1_ (*λ*), *P*_2_ (*λ*), and *P*_1_ (*λ*) to compensate for the power drift of the light source and the sensitivity variation of the MCT detector during the measurement. The first and last *P*_1_ (*λ*) were averaged from 1000 measurements, while the middle three *P*_2_ (*λ*), *P*_1_ (*λ*), and *P*_2_ (*λ*) were averaged from 2000 measurements. We then repeated the whole sequence once more. All *P*_1_ (*λ*) and *P*_2_ (*λ*) were averaged when put into Equation (1). Figure 9 shows the SDR spectral reflectance measured in turn by NIST and by KRISS, plotted in black and red lines, respectively. The reflectance value measured by NIST is traceable to the NIST primary reflectometer based on the absolute method [30]. The two results are consistent, within 1.2%. The NIST reported their standard uncertainty to be 1.6% (*k* = 1), and our standard uncertainty is about 2.18% (*k* = 1). The related uncertainty components of our measurement are tabulated in Table 1. Drift of the light source power and MCT sensitivity was monitored for 1 h, and their uncertainties were evaluated from the standard deviation of the results. The overall standard uncertainty includes ISW reflectance and diffusivity, vertical and horizontal shifts of the input beam, power drift of the light source, linearity of the MCT, sensitivity drift of the MCT, and repeatability.

**Figure 10:**
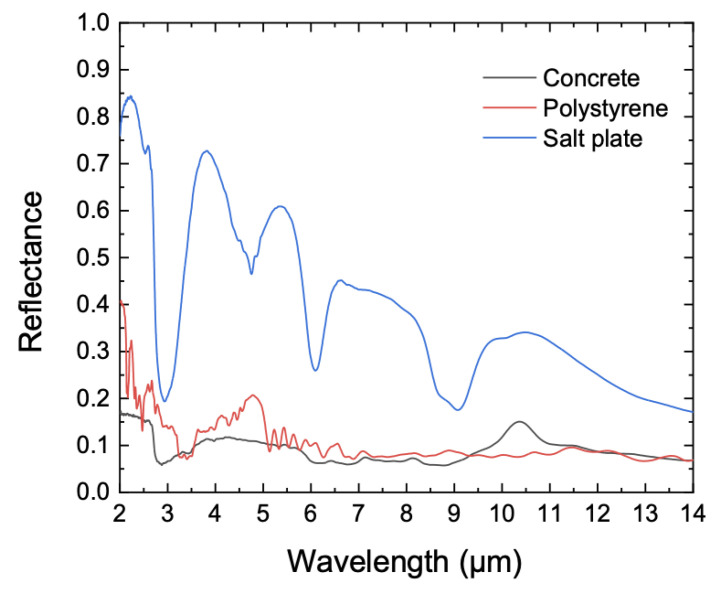
Spectral diffuse reflectance of three samples measured by the reflectometer for relative reflectance.

We then measured the spectral diffuse reflectance of various samples using the reflectometer for relative reflectance. The three samples were a concrete plate, a polystyrene plate, and a salt plate. According to the measurement procedure mentioned in Section 3, the spectral radiance of the FOV was measured when the sample was illuminated, *P_A_* (*λ*), and then when the SDR was illuminated, *P_B_* (*λ*). The measurement was repeated in the sequence *P_A_* (*λ*), *P_B_* (*λ*), *P_A_* (*λ*), *P_B_* (*λ*), and *P_A_* (*λ*) as in the absolute reflectance measurement. Also like the absolute measurement procedure, in this case the first and last *P_A_* (*λ*) were averaged from 1000 measurements, and the middle three *P_B_* (*λ*), *P_A_* (*λ*), *P_B_* (*λ*) were averaged from 2000 measurements, with the whole sequence repeated once more. All *P_A_* (*λ*) and *P_B_* (*λ*) were averaged when put into Equation (2). Figure 10 shows the spectral diffuse reflectances of the samples from 2 µm to 14 µm. The measurement uncertainty was evaluated as 2.99% (*k* = 1); the uncertainty components are tabulated in Table 2.

## 7. Conclusions

In conclusion, we built two reflectometers for spectral diffuse reflectance in the MIR range in directional-hemispherical geometry: one for absolute reflectance and one for relative reflectance. Each reflectometer was put in a vacuum chamber with an FTIR installed to obtain spectral radiance information. We also ran simulations to quantify the systematic uncertainties of each reflectometer using a ray-tracing method. The spectral diffuse reflectance scale of the SDR was realized by using the reflectometer for absolute reflectance, and its relative standard uncertainty was estimated to be 2.18% (*k* = 1). The measurement capability was validated by comparison with the NIST measurement result, which showed a very strong agreement within less than 1.2%. The spectral diffuse reflectance of three different SUTs was calibrated against the SDR using the reflectometer for relative reflectance, with a relative standard uncertainty of about 2.99% (*k* = 1).

## Figures and Tables

**Table 1:** Uncertainty factors and evaluated overall uncertainty for absolute reflectance measurement (ISW diffusivity is valid at 2 µm; PDF: probability distribution function; DOF: degree of freedom)

Description	Uncertainty Components	Standard Uncertainty	PDF	Sensitivity Coefficient	Contribution	DOF
ISW reflectance	*u_r_*(*R_SDR_*)_ISW_	1.6%	Standard	Ray-tracing simulation	0.2%	∞
Vertical shift	*u_r_* (*R_SDR_*)_vertical_	1 mm	Square	Ray-tracing simulation	0.1%	∞
Horizontal shift	*u_r_* (*R_SDR_*)_horizontal_	1 mm	Square	Ray-tracing simulation	0.5%	∞
ISW diffusivity	*u_r_* (*R_SDR_*)_diffusivity_	0.1	Standard	Ray-tracing simulation	0.5%	∞
Light source power drift	*u_r_* (*P*_1_)_source_	0.5%	Standard	0.95	0.47%	∞
	*u_r_* (*P*_2_)_source_				0.47%	
MCT linearity	*u_r_* (*P*_1_)_linear_	0.58%	Square	0.95	0.55%	∞
	*u_r_* (*P*_2_)_linear_				0.55%	
MCT sensitivity drift	*u_r_* (*P*_1_)_sensitivity_	0.87%	Square	0.95	0.82%	∞
	*u_r_* (*P*_2_)_sensitivity_				0.82%	
Repeatability	*u_r_* (*P*_1_)_rep_	1%	t	0.95	0.95%	4
	*u_r_* (*P*_2_)_rep_				0.95%	
Overall Uncertainty			Standard		2.18%	55.5

**Table 2:** Uncertainty factors and evaluated overall relative uncertainty for relative reflectance measurement (ISW diffusivity is valid at 2 µm; PDF: probability distribution function; DOF: degree of freedom)

Description	Uncertainty Components	Standard Uncertainty	PDF	Sensitivity Coefficient	Contribution (Relative)	DOF
SDR reflectance uncertainty	*u*_r_(*R*_SDR_)	2.07%	Standard	1	2.07%	∞
Light source power drift	*u*_r_(*P_A_*)_source_	0.5%	Standard	1	0.5%	∞
	*u*_r_(*P_B_*)_source_				0.5%	
MCT linearity	*u*_r_(*P_A_*)_linear_	0.58%	Square	1	0.58%	∞
	*u*_r_(*P_B_*)_linear_				0.58%	
MCT sensitivity drift	*u*_r_(*P_A_*)_sensitivity_	0.87%	Square	1	0.87%	∞
	*u*_r_(*P_B_*)_sensitivity_				0.87%	
Repeatability	*u_r_* (*P_A_*)_rep_	1%	t	1	1%	4
	*u_r_* (*P_B_*)_rep_				1%	
Overall Uncertainty			Standard		2.99	159.9

## Data Availability

Data available on request from the authors.
